# The LUNG SAFE study: a presentation of the prevalence of ARDS according to the Berlin Definition!

**DOI:** 10.1186/s13054-016-1443-x

**Published:** 2016-09-09

**Authors:** Giacomo Bellani, John G. Laffey, Tài Pham, Eddy Fan, On behalf of the LUNG SAFE Investigators and the ESICM Trials Group

**Affiliations:** 1 School of Medicine and Surgery, University of Milan-Bicocca, Monza, Italy; 2 Department of Emergency and Intensive Care, San Gerardo Hospital, Monza, Italy; 3 Departments of Critical Care Medicine, Keenan Research Centre for Biomedical Science, St Michael’s Hospital, University of Toronto, Toronto, Canada; 4 Departments of Anesthesia and Physiology, University of Toronto, Toronto, Canada; 5 Interdepartmental division of Critical Care Medicine, University of Toronto, Toronto, Canada; 6 Department of Medicine, University Health Network and Mount Sinai Hospital, Toronto, Canada; 7 Institute of Health Policy, Management and Evaluation, University of Toronto, Toronto, Canada

Villar et al. [1] suggest four methodological sources of “bias” that could have led to an overestimation of the acute respiratory distress syndrome (ARDS) incidence in the LUNG SAFE study [2]. First, they suggest that an “unvalidated algorithm” was used to classify patients with ARDS. We simply utilized the Berlin Definition for ARDS [3]. When the Berlin criteria were met, patients were classified as having ARDS—this is the “computer algorithm”. There was no need to “validate” this algorithm, since it simply verifies the presence or absence of Berlin ARDS criteria. The electronic CRF provided additional guidance, including what specifically was meant by “bilateral infiltrates”, and investigators were offered web-based training on chest X-ray image interpretation.

A second “bias” was the fact that some patients fulfilled the criteria for ARDS for less than 24 hours. The Berlin Definition does not include any time window for ARDS determination. We cannot prove or refute the authors’ statement that “Actual ARDS does not resolve in 24 h” [1]. In the absence of a gold standard, we cannot know what “actual ARDS” is.

A third suggested “bias” was the conduct of the study in winter months, an approach taken to minimize seasonal variation—a possible reason for differing ARDS prevalence estimates in prior reports. Contrary to the authors’ assertion, we did not extrapolate to the “year incidence of ARDS” but instead confined our estimates to a 4-week period prevalence [2].

Fourth, the authors suggest that the exclusion of ICUs not enrolling ARDS patients may constitute “the most biasing problem of all” [1]. This is incorrect. We excluded ICUs that did not enroll any ventilated patients (with or without ARDS). Including these centers would have not affected ARDS prevalence, because they would not have contributed to the numerator or the denominator of the prevalence calculations.

The authors also challenge the choice of classifying patients based on the worst PaO_2_/FiO_2_ with respect to the use of adjunctive treatments. The decision to classify patients in this way was made to better report the conditions (i.e., hypoxemia) that would have affected the decision-making of clinicians at the time when adjunctive measures were considered.

The authors also raise general concerns regarding the Berlin Definition of ARDS. The need for an improved definition of ARDS was not addressed in our study.

We trust that these clarifications address the authors’ concerns regarding the LUNG SAFE study, and thank them for their interest in our article.

## Abbreviations

ARDS, acute respiratory distress syndrome; CRF, case report form; PaO_2_/FiO_2_, ratio of arterial oxygen tension to inspired fraction of oxygen
